# Risk factors for identifying pneumocystis pneumonia in pediatric patients

**DOI:** 10.3389/fcimb.2024.1398152

**Published:** 2024-10-23

**Authors:** Chunyan Zhang, Zheng Li, Xiao Chen, Mengyuan Wang, Enhui Yang, Huan Xu, Shifu Wang

**Affiliations:** ^1^ Department of Microbiology Laboratory, Children’s Hospital Affiliated to Shandong University, Jinan, China; ^2^ Department of Clinical Microbiology, Shandong Provincial Clinical Research Center for Children’s Health and Disease, Jinan, China; ^3^ Department of Outpatient, 94201 Military Hospital, Jinan, China; ^4^ Department of Scientific Affairs, Vision Medicals Center for Infectious Diseases, Guangzhou, China

**Keywords:** pneumocystis pneumonia, metagenomic next-generation sequencing, receiver operating characteristic curve, area under the curve, pediatric

## Abstract

**Objectives:**

This study aimed to identify the risk factors and construct the diagnostic model associated with pneumocystis pneumonia (PCP) in pediatric patients.

**Methods:**

This retrospective observational study analyzed 34 cases of PCP and 51 cases of other types of pneumonia treated at Children’s Hospital Affiliated to Shandong University between January 2021 and August 2023. Multivariate binary logistic regression was used to identify the risk factors associated with PCP. Receiver operating characteristic curves and calibration plots were constructed to evaluate the diagnostic model.

**Results:**

Twenty clinical variables significantly differed between the PCP and non-PCP groups. Multivariate binary logistic regression analysis revealed that dyspnea, body temperature>36.5°C, and age<1.46 years old were risk factors for PCP. The area under the curve of the diagnostic model was 0.958, the *P*-value of Hosmer‐Lemeshow calibration test was 0.346, the R^2^ of the calibration plot for the actual and predicted probability of PCP was 0.9555 (*P*<0.001), and the mean Brier score was 0.069. In addition, metagenomic next-generation sequencing revealed 79.41% (27/34) and 52.93% (28/53) mixed infections in the PCP and non-PCP groups, respectively. There was significantly more co-infection with cytomegalovirus and *Streptococcus pneumoniae* in the PCP group than that in the non-PCP group (p<0.05).

**Conclusions:**

Dyspnea, body temperature>36.5°C, and age<1.46 years old were found to be independent risk factors for PCP in pediatric patients. The probability of co-infection with cytomegalovirus and *S. pneumoniae* in the PCP group was significantly higher than that in the non-PCP group.

## Introduction

Pneumocystis pneumonia (PCP) is a fungal infectious disease of the respiratory system caused by *Pneumocystis jirovecii*. Pneumocystis pneumonia is the most common opportunistic infection among patients with human immunodeficiency virus (HIV) infection. Recently, the prevalence of PCP in patients with HIV has decreased due to the use of highly effective antiretroviral therapy and prevention. However, with the increase in the number of patients with cancer, solid organ transplantation, and autoimmune diseases, along with advances in bronchoscopy and microbiological testing techniques, the morbidity and mortality of non-HIV-related PCP have significantly increased (Niu et al., 2023). In the early stage of *P. jirovecii* infection, patients may be asymptomatic. However, in the late stage, PCP progresses rapidly, leading to severe respiratory failure, poor prognosis, and high mortality rates (Liu et al., 2019).

The prognosis for pediatric patients with PCP largely depends on early, timely, and accurate diagnosis (Lu et al., 2022). Polymerase chain reaction (PCR) is more commonly used in the diagnosis of PCP, but the infection rate of *P. jirovecii* in pediatric patients is very low, and many children’s specialized hospitals do not carry out PCR methods to detect PCP (Liu et al., 2020). It has been reported that combining the detection of *P. jirovecii* sequences in bronchoalveolar lavage fluid (BALF) and peripheral blood by metagenomic next-generation sequencing (mNGS), with the patient’s clinical manifestations and computed tomography imaging features, can confirm the diagnosis of PCP, but only a few cases have been reported in the clinic (Zhang et al., 2019). In this study, we reviewed clinical data and mNGS reports from 85 children diagnosed with pneumonia, including 34 cases of PCP and 51 cases of other pneumonia types. We identified potential risk factors associated with PCP and developed a diagnostic model based on these factors. This model aims to serve as an early indicator of PCP infection risk in non-HIV patients.

## Methods

### Study design

A total of 34 children with PCP were retrospectively enrolled between January 2021 and August 2023. The diagnostic criteria (Donnelly et al., 2020; Lagrou et al., 2021; Huang et al., 2023)for PCP group were as follows: 1) Clinical symptoms with fever, cough, or shortness of breath; 2) Chest computerized tomography (CT) showed multiple rounds of glassy interstitial exudate, reticulated or solid shadows in both lungs on an chest computerized tomography; 3) The sequence of *P. jirovecii* was detected by mNGS; 4) The HIV test was negative. At the same time, 51 non-HIV patients who were admitted to the hospital with lung infections and diagnosed with non-PCP pneumonia as the non-PCP group during the same period were included. The clinical diagnosis of PCP and non-PCP was made by two senior respiratory specialists based on the clinical symptoms, laboratory findings, chest CT images, etiology of mNGS, and clinical response to treatment. The clinical data, including gender, age, underlying disease, clinical manifestations, CT imaging, mNGS test, and laboratory examinations, were collected from the patient’s medical records.

This study was approved by the Ethics Committee of the Children’s Hospital Affiliated to Shandong University (No. SDFE-IRB/P-2022017) and was conducted by the Declaration of Helsinki (revised 2013). The Ethics Committee of the Children’s Hospital Affiliated with Shandong University waived the need for individual informed consent for this retrospective analysis.

### Metagenomic next-generation sequencing

The DNA was extracted from BALF using a QIAamp^®^ UCP Pathogen DNA Kit (Qiagen), adhering to the manufacturer’s instructions. Human DNA was removed using Benzonase (Qiagen) and Tween 20 (Sigma). Total RNA was extracted with a QIAamp ^®^ Viral RNA Kit (Qiagen) and ribosomal RNA was removed with a Ribo-Zero rRNA Removal Kit (Illumina). cDNA was generated using reverse transcriptase and dNTPs (Thermos Fisher). Libraries were constructed for the DNA and cDNA samples using a NextEra XT DNA Library Prep Kit (Illumina, San Diego, CA). The library was purified, and magnetic beads selected the fragments. The library quality was assessed with a Qubit dsDNA HS Assay Kit followed by a High Sensitivity DNA kit (Agilent) on an Agilent 2100 Bioanalyzer. The library pools were then loaded onto an Illumina NextSeq CN500 sequencer for 75 cycles of single-end sequencing to generate approximately 20 million reads for each library. For negative controls, we also prepared sterile deionized water in parallel with each batch to serve as a non-template control, using the same protocol.

High-quality sequencing data were generated by removing low-quality and short (length < 40 bp) reads, followed by computational subtraction of human host sequences mapped to the human reference genome (hg38 and YH sequences) using Burrows-Wheeler alignment. The remaining data obtained by removing low-complexity reads were classified by simultaneous alignment to four microbial genome databases, consisting of viruses, bacteria, fungi, and parasites. The classification reference databases were downloaded and optimized from public databases such as NCBI and GenBank. In the end, the multi-parameters of Species in the microbial genome databases were calculated and exported, and professionals with microbiology and clinical backgrounds interpreted the results.

### Statistical analysis

The data were analyzed using IBM SPSS 26.0 statistical software. Normally distributed continuous data are presented as the mean ± SD and were compared using a t-test. Continuous data that did not fit a normal distribution are presented as the median (Q1, Q3) and were compared using the Mann−Whitney U test. The discontinuous data are presented as n (%) and were compared using the χ^2^ test or Fisher’s test. Univariate logistic regression was used to identify the risk factors associated with PCP. Variables with *P*-value ≤0.1 were further analysed via multiple logistic regression, and variables with *P*-value < 0.05 were used to construct the diagnostic model. The diagnostic model was evaluated using receiver operating characteristic (ROC) curves and Hosmer-Lemeshow goodness-of-fit tests. GraphPad Prism 9.3 was utilized to create figures, including ROC curves and forest plots.

## Results

### Patient characteristics

As shown in **Table 1**, the 20 clinical variables showed significant differences between the PCP and non-PCP groups. The PCP group had 21 (61.76%) boys, while the no-PCP group had 31 (60.78%) boys, with no significant difference observed. The median age was 0.58 years old in the PCP group and 5.33 years in the non-PCP group, and the difference was remarkable (*P <*0.001). In terms of underlying diseases, the PCP group had significantly higher rates of surgery (50.00% vs 9.80%, *P <*0.001), tracheal dysplasia (55.88% vs 13.72%, *P <*0.001), and premature delivery (35.29% vs 5.88%, *P <*0.001) compared to the non-PCP group. Regarding clinical manifestations, dyspnea (94.12% vs 13.73%, *P <*0.001), body temperature (37.40°C vs 36.50°C, *P* =0.002), heart rate (135 beats vs 105 beats, *P <*0.001), and respiratory rate (35 beats vs 28 beats, *P <*0.001) were significantly greater in the PCP groups than in the non-PCP group.

**Table 1:** Patient’ characteristics, laboratory findings and CT images of PCP and non-PCP pediatric patients.

Clinical information	Total	PCP (N=34)	non-PCP (N=51)	*P*-value
**Gender(male)**	52 (61.18%)	21 (61.76%)	31 (60.78%)	0.761
**Age**	2.00(0.42, 7.00)	0.58 (0.27, 1.00)	5.33 (1.625~8.00)	<0.001
Underlying diseases				
Congenital heart diseases	9 (10.59%)	5 (14.71%)	4 (7.84%)	0.304
Post-surgery	22 (25.88%)	17 (50.00%)	5 (9.80%)	<0.001
Pulmonary artery sling	2 (2.35%)	2 (5.88%)	0	0.15
Tracheal dysplasia	26 (30.58%)	19 (55.88%)	7 (13.72%)	<0.001
Malignant tumor	5 (5.88%)	4 (11.76%)	1 (1.96%)	0.074
Immunocompromised	13 (15.29%)	8 (23.53%)	5 (9.80%)	0.072
Premature birth	15 (17.64%)	12 (35.29%)	3 (5.88%)	<0.001
Clinical manifestations				
Fever	63 (74.12%)	26 (76.47%)	37 (72.55%)	0.498
Dyspnea	39 (45.88%)	32 (94.12%)	7 (13.73%)	<0.001
Cough	76 (89.41%)	30 (88.23%)	46 (90.20%)	1
Body temperature	36.50 (36.20, 37.70)	37.40 (36.45, 38.43)	36.50 (36.20, 36.80)	0.002
Heart rate	120 (100.00, 136.00)	135 (121.50, 150.50)	105 (98.00, 122.00)	<0.001
Respiratory rate	34 (25.00, 40.00)	35 (30.00, 40.50)	28 (24.00, 36.50)	0.005
Laboratory findings				
White blood cells (10^9^/L)	9.79 ± 4.94	9.67 ± 4.88	9.87 ± 5.03	0.854
Neutrophils (%)	56.69 ± 21.39	53.78 ± 23.04	58.55 ± 20.26	0.313
Lymphocyte (%)	34.04 ± 19.63	37.23 ± 21.3	31.99 ± 18.39	0.226
Hemoglobin (g/L)	117 (101.00, 128.00)	108 (93.25, 119.75)	121 (112.00, 129.00)	0.004
Platelets (10^9^/L)	342.67 ± 164.40	305.21 ± 141.50	366.70 ± 174.61	0.089
Albumin (g/L)	38.59 ± 6.22	39.50 ± 6.49	38.00 ± 6.03	0.017
Aspartate aminotransferase (U/L)	36.00 (28.00, 52.00)	43.00 (31.00, 65.25)	34.00 (27.00, 45.50)	0.010
Alanine aminotransferase (U/L)	21.00 (14.00, 39.00)	24 (16.75, 39.25)	20.00 (13.00, 37.50)	0.148
Creatinine (μmol/L)	24.00 (16.00, 32.00)	17.00 (15.75, 23)	28.00 (20.50, 33.00)	0.001
Urea (mmol/L)	3.80 (2.42, 4.60)	3.46 (2.32, 4.70)	3.87 (2.92, 4.41)	0.503
Direct bilirubin (μmol/L)	3.30 (2.60, 5.10)	3.45 (2.65, 7.13)	3.20 (2.40, 4.30)	0.199
Total bilirubin (μmol/L)	7.30 (4.60, 12.40)	8.05 (4.75, 22.83)	6.70 (4.45, 10.10)	0.115
Total protein (g/L)	61.20 (55.70, 67.00)	59.00 (52.05, 64.10)	62.10 (59.05, 67.05)	0.027
Lactate dehydrogenase (U/L)	288.00 (225.00, 365.00)	302.50 (226.25, 401.00)	283.00 (224.50, 362.50)	0.528
Procalcitonin (ng/ml)	0.12 (0.07, 0.25)	0.16 (0.09, 0.60)	0.12 (0.07, 0.18)	0.027
C-reactive protein (mg/L)	4.49 (0.50, 38.75)	3.92 (0.50, 66.18)	4.49 (0.50, 30.25)	0.955
Prothrombin time (S)	12.20 (11.50, 13.20)	12.25 (11.45, 13.60)	12.00 (11.45, 13.00)	0.428
Activated thrombin time (S)	28.75 (24.5, 35.60)	30.55 (25.93, 39.73)	27.8 (23.65, 32.65)	0.020
D-dimer (mg/L)	0.78 (0.40, 1.94)	0.73 (0.39, 1.54)	0.78 (0.40, 2.02)	0.557
CD3 (%)	61.37 (54.32, 67.49)	61.37 (50.32, 66.28)	61.37 (57.29, 68.91)	0.097
CD8 (%)	25.01 (19.28, 28.38)	20.24 (13.41, 25.10)	25.01 (24.39, 29.89)	0.001
CD4 (%)	33.64 (28.22, 36.72)	33.64 (24.11, 39.4)	33.64 (29.26, 36.04)	0.847
Computed Tomography images				
Bilateral lesions	73 (85.88%)	34 (100.00%)	39 (73.47%)	0.001
Pleural effusion	23 (27.05%)	3 (8.82%)	20 (39.22%)	0.003
Ground-glass opacity	12 (14.11%)	11 (32.35%)	1 (1.96%)	<0.001
Emphysema	38 (44.71%)	13 (38.23%)	25 (49.02%)	0.412
**Duration of hospital stay**	12.00 (9.00, 17.00)	14.00 (9.75, 21.25)	12.00 (9.00, 15.00)	0.178
**Duration from onset to admission**	13.00 (6.00, 30.00)	12.50 (5.00, 74.00)	13.00 (6.00, 20.00)	0.188
**Mechanical ventilation**	19.00 (22.35%)	17.00 (50.00%)	2.00 (3.92%)	<0.001
Outcome				
28-day death	3 (3.53%)	2 (5.88%)	1 (1.96%)	0.558
Death of discharge	2 (2.35%)	2 (5.88%)	0	0.158

PCP, Pneumocystis pneumonia.

There were no remarkable differences in routine blood parameters, such as white blood cell count, neutrophils count, or lymphocyte percentage between the two groups. However, in terms of blood biochemistry, hemoglobin (108 vs 121, p=0.004), creatinine (17 vs 28, *P* =0.001), and total protein (59.00 vs 62.10, *P* =0.27) were significantly decreased in the PCP group. On the other hand, the levels of albumin (39.50 vs 38.00, *P* =0.017), aspartate aminotransferase (43 vs 34, *P* =0.01), procalcitonin (0.16 vs 0.12, *P* =0.27), and activated partial thromboplastin time (30.55 vs 27.80, *P* =0.02) were significantly increased in the PCP group. Additionally, the PCP group exhibited a significant increase in CD8+ T lymphocytes (20.24 vs 25.01, *P* =0.001). Computerized tomography images revealed that bilateral lesions (100.00% vs 73.60%, *P* =0.001) and ground glass shadow (32.40% vs 1.90%, *P <*0.001) were more common in the PCP group, whereas pleural effusion (8.80% vs 26.40%, *P* =0.003) was more prevalent in the non-PCP group. Furthermore, mechanical ventilation (50% vs 3.80%, *P <*0.001) was significantly more common in the PCP group.

### Risk factors for PCP

**Figure 1:**
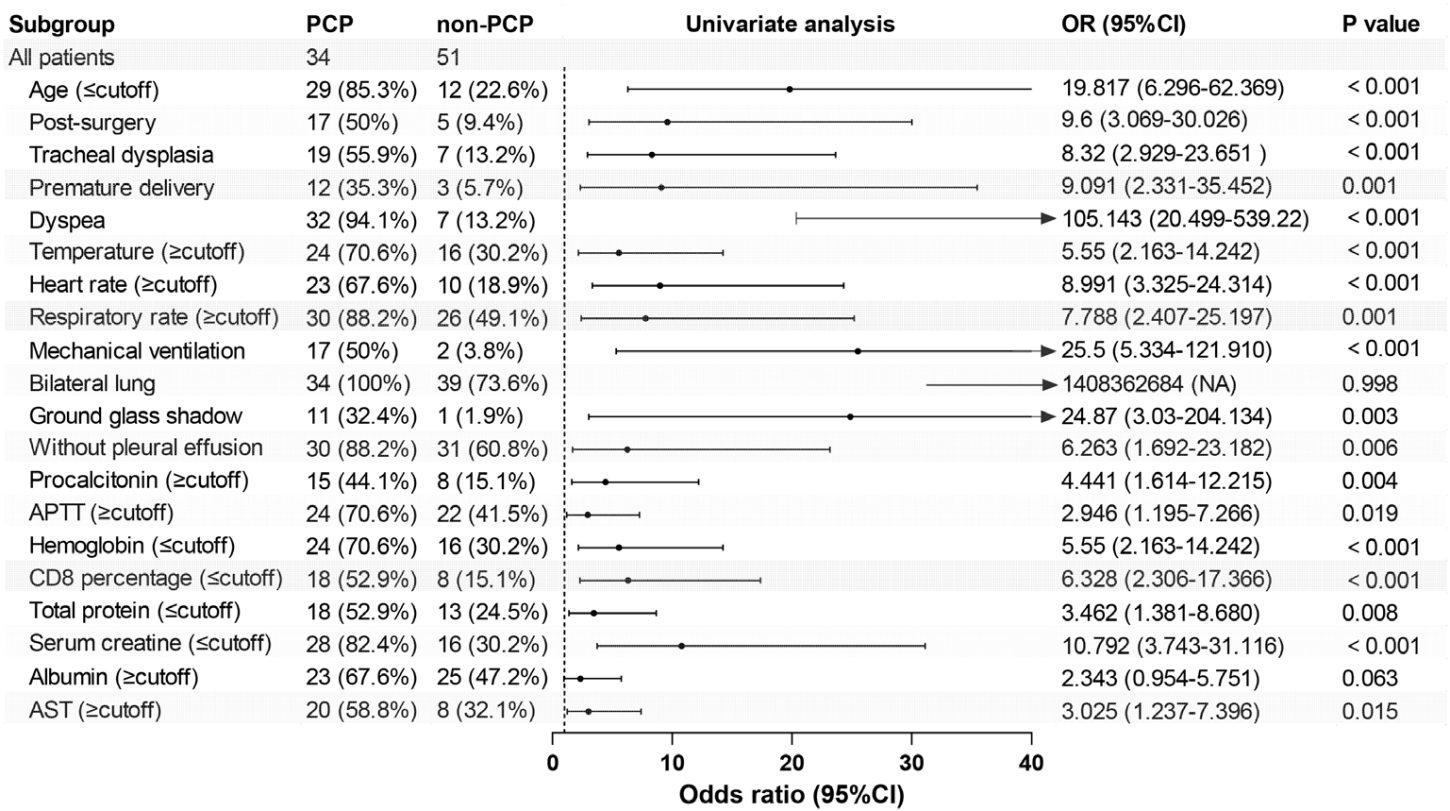
Forest map of 20 risk factors identified in the univariate logistic analysis for the PCP group. APTT, activated partial thromboplastin time; AST, aspartate aminotransferase.

As depicted in **Table 2**, we analyzed the ability of the above-mentioned 20 clinical variables to predict PCP using the ROC curves and binary logistic regression. By applying the principle of maximum sensitivity and specificity, ROC curves allowed us to determine the cut-off values for continuous variables and convert them into categorical variables (Nahm, 2022). Based on the ROC curves, the thresholds for age, body temperature, heart rate, CD8^+^ T lymphocytes, and serum creatinine were found to be 1.46, 36.65, 127.00, 20.84, and 24.50, respectively. The areas under the ROC curves for dyspnea, mechanical ventilation, post-surgery, tracheal dysplasia, age, temperature, heart rate, CD8^+^ T lymphocytes, and serum creatinine all exceeded 0.70 (0.905, 0.731, 0.703, 0.713, 0.752, 0.700, 0.794, 0.711, and 0.719, respectively; 95% confidence intervals (CIs) were 0.834-0.975, 0614-0.848, 0.584-0.822, 0.596-0.830, 0.641-0.862, 0.579-0.821, 0.691-0.896, 0.579-0.821, 0.691-0.896, 0.596-0.825, and 0.579-0.841; all *P*-values were less than 0.05 (<0.001, <0.001, 0.001, 0.001, <0.001, 0.002, <0.001, 0.001, 0.001, respectively). Subsequently, univariate binary logistic regression analysis was used to screen for risk factors. **Table 2** and **Figure 1** illustrated those 18 variables, excluding bilateral lung lesions and albumin, the serum ALB concentration, were significantly different, with *P*-values lower than 0.05.

**Table 2:** ROC curve and univariate binary logistic regression analysis of risk factors for PCP.

Factors	ROC Curve					Binary logistic regression analysis			
	Cut-off	Youden’s index	AUC	95% CIs	*P*-value	Coefficient	OR	95% CIs	*P*-value
**Bilateral lung lesions**	NA	0.264	0.632	0.517-0.747	0.038	21.066	1408362684	NA	0.998
**Ground glass shadow**	NA	0.305	0.652	0.528-0.777	0.017	3.214	24.870	3.030-204.134	0.003
**Without Pleural effusion**	NA	0.289	0.645	0.529-0.760	0.023	1.835	6.263	1.692–23.182	0.006
**Dyspnea**	NA	0.809	0.905	0.834-0.975	<0.001	4.655	105.143	20.499–539.220	<0.001
**Mechanical ventilation**	NA	0.462	0.731	0.614-0.848	<0.001	3.239	25.500	5.334-121.910	<0.001
**Post-surgery**	NA	0.406	0.703	0.584-0.822	0.001	2.262	9.600	3.069-30.026	<0.001
**Tracheal dysplasia**	NA	0.427	0.713	0.596-0.83	0.001	2.119	8.320	2.929–23.651	<0.001
**Premature delivery**	NA	0.296	0.648	0.524-0.772	0.02	2.207	9.091	2.331–35.452	0.001
**Age (y)**	1.46	0.627	0.752	0.641-0.862	<0.001	2.987	19.817	6.296-62.369	<0.001
**Body Temperature (°C)**	36.65	0.404	0.700	0.579-0.821	0.002	1.714	5.550	2.163–14.242	<0.001
**Heart rate (/min)**	127.00	0.488	0.794	0.691-0.896	<0.001	2.196	8.991	3.325–24.314	<0.001
**Procalcitonin (ng/mL)**	0.24	0.290	0.641	0.517-0.765	0.027	1.491	4.441	1.614–12.215	0.004
**activated partial thromboplastin time (S)**	28.25	0.291	0.649	0.528-0.769	0.02	1.108	2.946	1.195–7.266	0.019
**Respiratory rate (/min)**	29.00	0.392	0.679	0.564-0.794	0.005	2.053	7.788	2.407-25.197	0.001
**Hemoglobin (g/L)**	114.50	0.404	0.683	0.558-0.808	0.04	1.714	5.550	2.163-14.242	<0.001
**CD8 percentage (%)**	20.84	0.378	0.711	0.596-0.825	0.001	1.845	6.328	2.306-17.366	<0.001
**Total protein (g/L)**	59.05	0.284	0.641	0.520-0.762	0.027	1.242	3.462	1.381-8.680	0.008
**Serum creatine (μmol/L)**	24.50	0.522	0.719	0.597-0.841	0.001	2.379	10.792	3.743-31.116	<0.001
**Albumin (g/L)**	37.55	0.205	0.585	0.457-0.713	0.183	0.851	2.343	0.954-5.751	0.063
**Aspartate aminotransferase (U/L)**	38.50	0.267	0.664	0.548-0.779	0.010	1.107	3.025	1.237-7.396	0.015

PCP, Pneumocystis Pneumonia; ROC Curve, receiver operating characteristic curves; AUC, area under the curve; CIs, confidence intervals; OR, odds ratio.

### A diagnostic model for PCP

**Figure 2:**
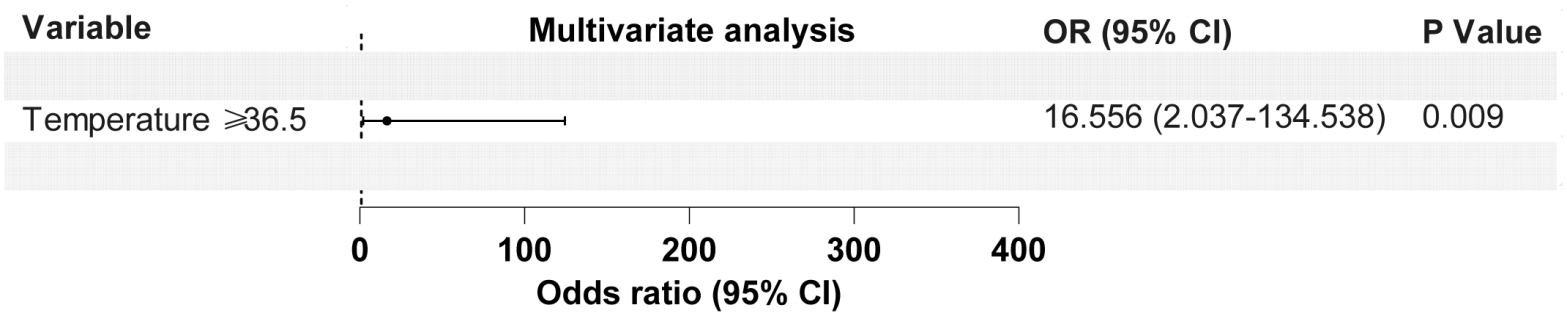
Forest map of 3 risk factors identified by the multivariate logistic analysis for the PCP group.

Based on the results of the ROC curve and univariate logistic analysis, nine variables, including dyspnea, mechanical ventilation, post-surgery, tracheal dysplasia, age, temperature, heart rate, CD8^+^ T lymphocytes, and serum creatinine, were subjected to multivariate logistic regression analysis. As shown in **Table 3** and **Figure 2**, dyspnea, age, and body temperature were found to be significantly associated with PCP, with odds ratios of 52.003, 10.233, and 16.556; 95% CIs of 7.023-385.037, 1.216-86.147, and 2.037-134.538; and *P*-values of <0.001, 0.032, and 0.009, respectively.

**Table 3:** Multivariate logistic regression analysis of risk factors for PCP.

Risk factors	B	S. E	Wald χ2	*P*-value	*OR* value	95% CIs
Dyspnea	3.951	1.021	14.963	<0.001	52.003	7.023-385.037
Age <1.46	2.326	1.087	4.578	0.032	10.233	1.216-86.147
Body Temperature >36.5 °C	2.807	1.069	6.894	0.009	16.556	2.037-134.538

PCP, Pneumocystis Pneumonia; B coefficient; S.E. standard error; OR odds ratio; CIs confidence intervals; Wald, Wald χ2.

**Figure 3:**
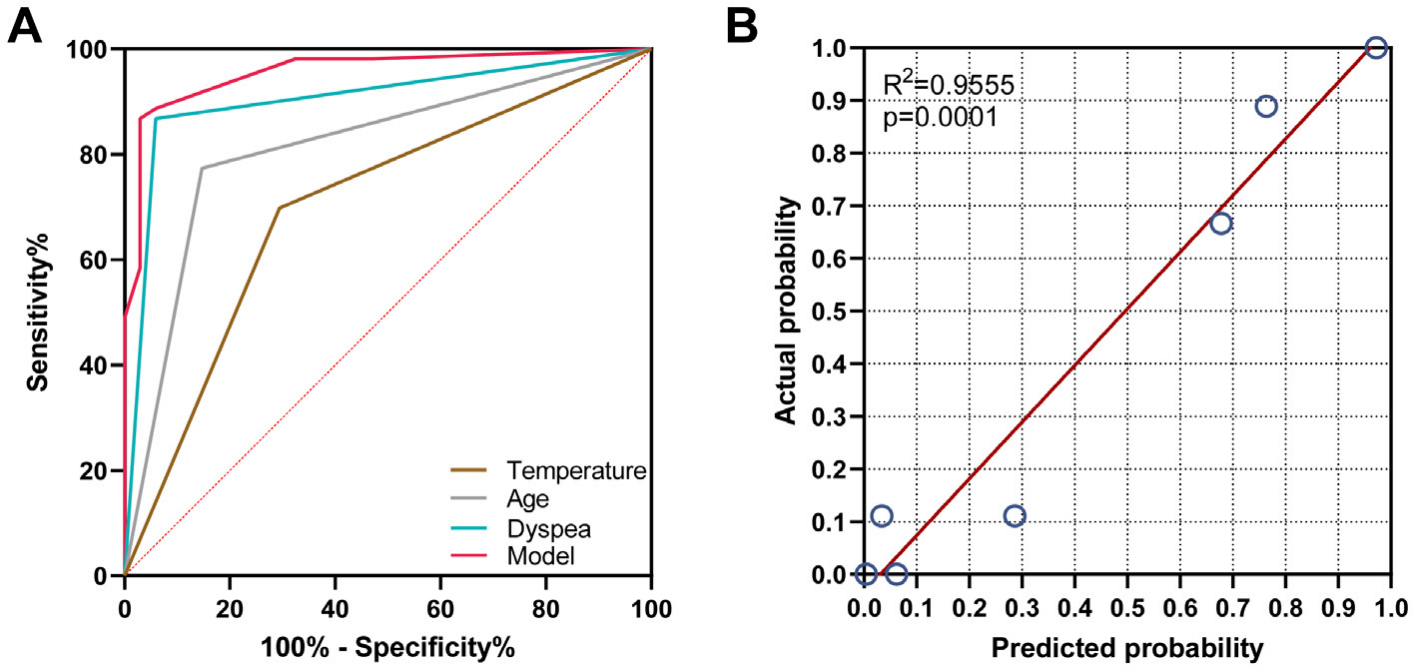
Validation of the model for predicting PCP probability. **(A)** The area under the receiver operating characteristic curve was 0.958, 0.905, 0.813, and 0.702 for the model group, dyspnea group, and age and body temperature group, respectively. **(B)** The calibration plot indicated that the predicted probability of PCP had a moderate agreement with the actual observed outcome (R^2^ = 0.9555, *P*<0.001).

The predictive accuracy for PCP as measured by the ROC curves (**Figure 3A**), yielded an area under the curve (AUC) of 0.958 (95% CI: 0.916–0.999, *P <*0.001). The Hosmer‐Lemeshow calibration test was significant (*P* =0.346), and the calibration plot (**Figure 3B**) for predicting PCP showed moderate agreement between the actual observed outcome and the prediction (R^2^ = 0.9555, *P <*0.001). Overall, the prediction performance was good, with a mean Brier score of 0.069 (95% CI: 0.032–0.104).

### Diagnostic performance of mNGS for mixed infections

**Figure 4:**
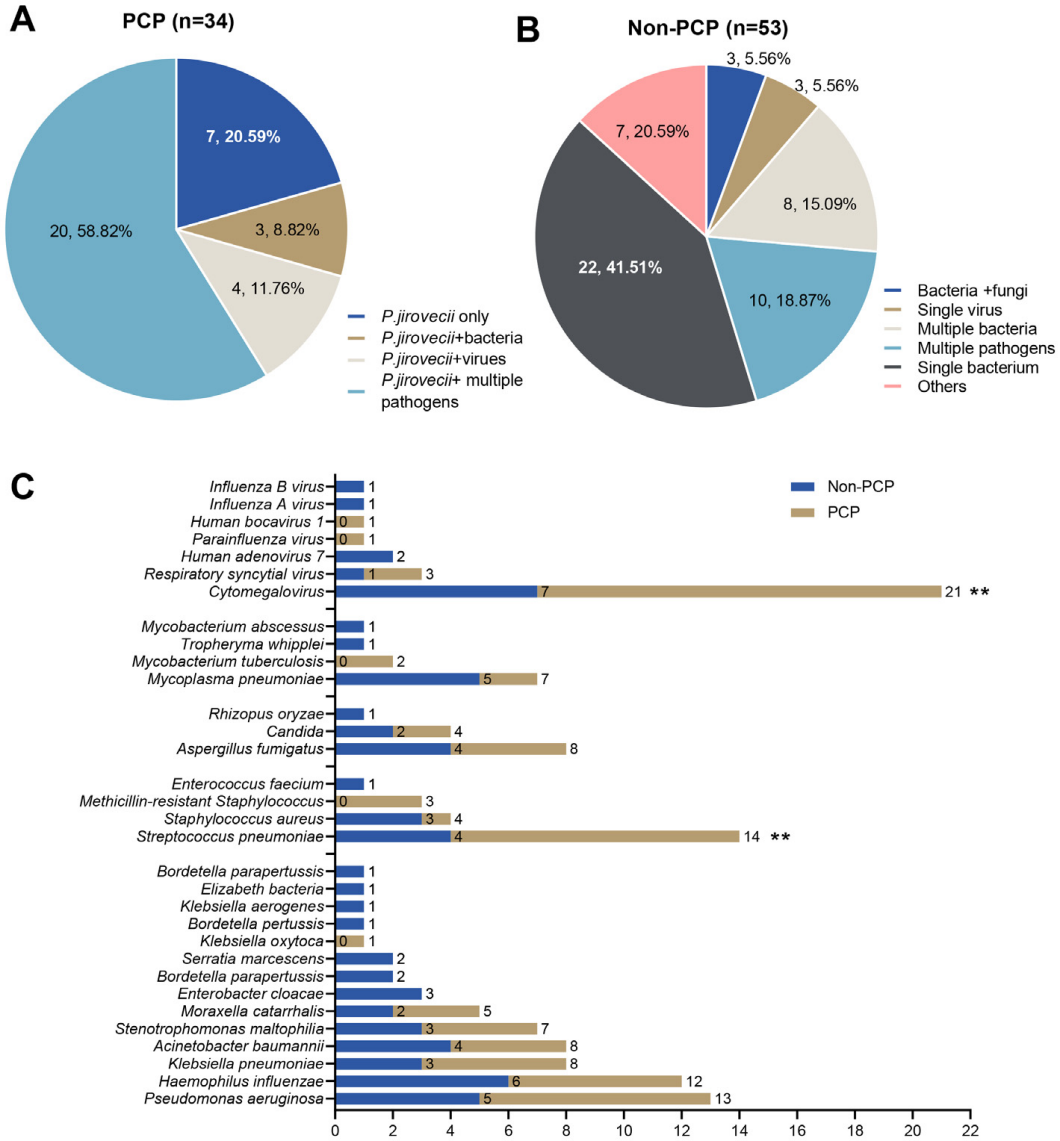
**(A)** The mixed infections were identified by mNGS in the PCP group. **(B)** The mixed infections were identified by mNGS in the non-PCP group. **(C)** Comparative analysis of major co-pathogenic in PCP group and non-PCP group (** Indicates p<0.01).

The mixed infections were identified in 79.41% of patients in the PCP group and 52.93% of those in the non-PCP group using mNGS (**Figures 4A, B**). In the PCP group, the major co-pathogens associated with *P. jirovecii*, included cytomegalovirus (CMV), *S. pneumoniae*, *Pseudomonas aeruginosa*, and *Haemophilus influenzae* (**Figure 4C**). The study using mNGS revealed a significantly higher rate of co-infection with CMV (41.18% vs 13.21%, *P* =0.003) and *S. pneumoniae* (29.41% vs 7.55%, *P* =0.007) in the PCP group (**Figure 4C**).

## Discussion

*P. jirovecii* is an opportunistic pathogen that is extremely rare in children and often becomes a pathogen when the host’s immune function is compromised, leading to severe lung infections. The clinical symptoms of the majority of patients with PCP were nonspecific. Due to the uncultivable nature of *P. jirovecii in vitro*, the gold standard for diagnosing PCP is the presence of characteristic encapsulated or trophozoite bodies in specimens from the lower respiratory tract (Huang et al., 2024). However, the low load of *P. jirovecii* in the lower respiratory tract of the patients and the reliance on the ability to examine physicians significantly hinder the early diagnosis, leading to misdiagnosis and underdiagnosis (Delliere et al., 2020; McDonald et al., 2024).

In this retrospective study, we developed a new diagnostic model for PCP using non-invasive and easily accessible clinical indicators. This diagnostic model demonstrated reliable discriminatory power in assessing the probability of PCP (Zhou and Aitken, 2023). It is expected to assist clinicians in early and rapid bedside screening for PCP in children, ultimately improving the prognosis of these patients in the future (Lagrou et al., 2021). We first conducted univariate logistic analysis and ROC curve analysis on 20 clinical variables from 85 patients with severe pneumonia. Among these nine variables exhibited a *P*-value <0.05 in the logistic analysis and an AUC>0.7 in the ROC curve, leading to their further inclusion in multivariate binary logistic regression. Subsequently, dyspnea, age<1.46 years, and temperature>36.5 were identified as the risk factors in the multivariate logistic analysis. Upon combining these three factors, the diagnostic model achieved an AUC of 0.958. The *P*-value of the Hosmer‐Lemeshow calibration test was 0.346, the R^2^ of the calibration plot for the actual and predicted probability of PCP was 0.9555 (p<0.001), and the mean Brier score was 0.069 (95% CI: 0.032–0.104), demonstrating good overall prediction performance.

Various specimens, such as blood, sputum, nasopharyngeal secretions, and BALF, can be utilized for microbiological testing. Among these methods, BALF is particularly effective in diagnosing PCP (Guegan and Robert-Gangneux, 2019; Georges et al., 2020). Various laboratory detection methods for PCP exist, including microscopic, serological, and imaging examinations. However, these methods have a high false-negative rate in early infection. Tissue biopsy, while effective, is an invasive procedure with associated risks and may not be suitable for all patients. In recent years, PCR detection technology has advanced significantly, with the emergence of molecular detection methods that offer high specificity and sensitivity in detecting specific target sequences. Despite these advancements, these technologies still have limitations in detecting rare or unknown pathogens. Due to the atypical clinical manifestations and the high prevalence of mixed infections in PCP patients, the mNGS assay offers direct detection of microorganism sequences in the clinical samples with shorter turn-around time, high sensitivity, and notably high detection rates for mixed bacterial, viral, fungal, and parasitic infections (Chiu and Miller, 2019; Wang et al., 2019; Zhang et al., 2021; Wang et al., 2022; Chang et al., 2023). Consequently, mNGS has demonstrated excellent performance in PCP diagnosis and co-pathogen detection (Jiang et al., 2021).

In our study, we observed mixed infections in 79.41% and 52.93% of patients in the PCP and non-PCP groups, respectively. Cytomegalovirus, *S. pneumoniae*, *P. aeruginosa*, and *H. influenzae* were the major co-pathogens in PCP patients, with significantly higher detection rates of CMV and *S. pneumoniae* in PCP compared to non-PCP patients (*P* < 0.005). Cytomegalovirus infection, typically preceding or concurrent with PCP, represents a risk factor for PCP and can significantly increase the morbidity and mortality of PCP in solid organ transplant recipients (Faure et al., 2017; Hosseini-Moghaddam et al., 2018). Infection with CMV has been suggested to be the most common cause of PCP and may be linked to immunosuppression by suppressing the function of helper T-cells and antigen-presenting cells, indicating severe immunosuppression (Lee et al., 2017). In terms of PCP co-infection, *S. pneumoniae* infection ranks second, primarily colonizing the nasopharynx of children (especially those under five years of age). In states of reduced immune function, it can proliferate rapidly and spread to other parts of the body, leading to infection (Morilla et al., 2021).

In conclusion, age<1.46 years, dyspnea, and temperature>36.5°C are identified as risk factors for PCP in non-HIV patients, and the diagnostic model based on these three factors demonstrated good predictive diagnostic value for PCP infection. The technology of mNGS technology plays a crucial role in confirming the diagnosis of PCP, especially for identifying mixed infections involving multiple pathogens, exhibiting strong diagnostic performance. However, among 1481 patients tested for mNGS, only 34 positive children were detected in this study, and the positive rate was only 2.30%. Aware of the limited incidence of PCP infection in children and currently few studies published focus on children with PCP who are not infected with HIV, in order to attract the attention of global pediatric experts on PCP in non-HIV, we first need to report the small size of study population. In the future, our team needs to accumulate clinical cases for another 3-5 years. Combining we create Chinese children’s bacteria and fungus resistance monitoring network member unit (https://www.etyy.com/respro.html), later we will have a longer period of clinical big queue (such as expanding time, try using multicenter retrospective). As well as prospective ways to expand the number of children) to validate, while conducting more in-depth subgroup analyses.

## Data Availability

The datasets generated during or analyzed during the current study are not publicly available but are available from the corresponding author upon reasonable request.
